# Duration of Hepatitis B Vaccine-Induced Protection among Medical Students and Healthcare Workers following Primary Vaccination in Infancy and Rate of Immunity Decline

**DOI:** 10.3390/vaccines10020267

**Published:** 2022-02-10

**Authors:** Nanthida Phattraprayoon, Jirapa Kakheaw, Kamonwan Soonklang, Kunsuda Cheirsilpa, Teerapat Ungtrakul, Chirayu Auewarakul, Nithi Mahanonda

**Affiliations:** 1 Princess Srisavangavadhana College of Medicine, Chulabhorn Royal Academy, Bangkok 10210, Thailand; nanthida.pha@cra.ac.th (N.P.); chirayu.aue@cra.ac.th (C.A.); 2 Chulabhorn Hospital, Chulabhorn Royal Academy, Bangkok 10210, Thailand; jirapa.pra@pccms.ac.th (J.K.); kunsuda.che@pccms.ac.th (K.C.); nithi.mah@cra.ac.th (N.M.); 3 Centre of Learning and Research in Celebration of HRH Princess Chulabhorn’s 60 Birthday Anniversary, Chulabhorn Royal Academy, Bangkok 10210, Thailand; kamonwan.soo@cra.ac.th

**Keywords:** hepatitis B, primary vaccination, healthcare workers, rate of immunity decline

## Abstract

Since the introduction of hepatitis B virus (HBV) vaccines, the numbers of HBV infections and complications have significantly decreased. However, the evidence on whether primary vaccination of infants confers lifelong immunity varies. We aimed to assess long-term immunity among healthcare workers and medical students, and the rate of decline of HBV surface antigen antibodies (anti-HBs). Hepatitis B status among participants born after 1 January 1992 was reviewed at Chulabhorn Royal Academy, Thailand. Participants were stratified by intervals since primary vaccination. HBV immunity was determined and analyzed as anti-HBs decline rate in participants with multiple follow-ups. A total of 464 participants were analyzed, with a median age of 23. Protective immunity against HBV (anti-HBs ≥ 10 mIU/mL) at 16–20, 21–25 and 26–28 years post-primary vaccination was 28%, 51.7% and 60%, respectively. The overall declining rate of anti-HBs was −42.39 mIU/mL per year. Participants with anti-HBs levels of >100–1000 mIU/mL at baseline had a faster decline rate than those with anti-HBs levels of 10–100 mIU/mL. Primary vaccination may not provide lifelong protection since HBV immunity deteriorates over time. Individuals with higher initial HBV immunity levels may experience a faster decline rate.

## 1. Introduction

Hepatitis B virus (HBV) infection results in acute and chronic liver disease and increases the risk of developing cirrhosis and hepatocellular carcinoma (HCC). It spreads via infected blood, bodily fluids and sexual contact. According to the World Health Organization (WHO), 257 million people worldwide are infected with HBV [1] or have tested positive for hepatitis B surface antigen (HBsAg), with 15% of those infected living in the WHO’s South-East Asia Region (SEAR). In 2015, the WHO reported that HBV-related diseases such as cirrhosis and HCC claimed 887,000 lives worldwide. Each year, the SEAR is estimated to lose 296,000 people to hepatitis B [1,2,3], mostly from HBV-related chronic liver cirrhosis and liver cancer [4].

Efficacious hepatitis B vaccines, now widely administered to infants, have resulted in significantly reduced new chronic HBV infections. In 2015, HBV transmission in the first five years of life decreased significantly, as indicated by a decrease in the percentage of children infected with HBV [5]. In 1992, Thailand implemented a national policy of immunizing all newborns with a universal monovalent HBV vaccine, adding this vaccine to the expanded program for immunization (EPI) and ensuring all children born after this date are vaccinated against HBV [6]. The vaccine is given in three doses: at birth, 2 and 6 months of age [7,8].

The long-term immune response following HBV vaccination in infancy has been studied in several countries. Antibodies against HBV were detected at high levels in some studies [9,10,11,12] and booster vaccination was therefore not recommended. However, similar studies involving long-term evaluations have demonstrated a decline in antibody levels in individuals who received HBV vaccination during infancy, and revaccination was recommended [13,14,15].

Long-term protective immunity (antibodies against hepatitis B surface antigen (anti-HBs) ≥ 10 mIU/mL) has been controversial among individuals who received the HBV vaccine as infants. In Thailand, Poovorawan et al. demonstrated long-term persistence of antibodies and immune memory against HBV following a four-dose HBV vaccine and single dose of HBV immunoglobulin at birth for up to 20 years after vaccination in participants born to hepatitis B surface antigen (HBsAg)-positive mothers [11]. In contrast, Posuwan et al. reported that of medical students born to HBV-infected mothers, 93.1% receiving either the diphtheria-tetanus-pertussis plus HBV vaccine or the combined diphtheria-tetanus-pertussis with HBV vaccine between birth and 6 months of age, in addition to the monovalent HBV vaccine at 1 month post-birth, developed insufficient protective anti-HBs antibodies during young adulthood [16].

Medical students and healthcare workers (HCWs) in hospitals are at risk of occupational exposure to HBV. Despite complete HBV vaccination, vaccine-induced antibody persistence may decline over time. It remains debatable whether to administer a booster dose to these high-risk groups if their anti-HBs levels fall below the protective threshold [17]. Our institute’s policy requires HBsAg and anti-HBs screening of all medical students and HCWs who begin studying/working at our institute, as well as annual HBsAg and anti-HBs surveillance to ensure that all HCWs have protective immunity. As such, our objective was to determine the seroprevalence of anti-HBs at various time points following primary vaccination during infancy as part of the EPI program and the rate of decline in protective anti-HBs in HCWs.

## 2. Materials and Methods

### 2.1. Expanded Immunization Program (EPI) in Thailand

Thailand’s Ministry of Public Health introduced national HBV vaccination in 1992, mandating routine HBV vaccination for all newborns at birth, 2 and 6 months of age. The first HBV vaccination was monovalent, followed by a single or combination vaccine series, with all infants receiving three to four doses of vaccine [18]. The hepatitis B vaccine coverage increased from 88% in 1995, to 96% in 2003 and 99.4% in 2013 [19].

### 2.2. Study Design and Methodology

A retrospective study was conducted by considering subjects who began training or working at Chulabhorn Royal Academy, Thailand, from January 2012 to August 2021. Students and HCWs born after 1 January 1992, who were tested for hepatitis B status at the time they started working or training, were included in the study.

We reviewed medical records for test results regarding anti-HBs and HBsAg, and individuals whose medical records indicated they had received an HBV booster dose or were positive for HBsAg were excluded. The presence or absence of HBsAg was examined using Elecsys HBsAg II kit (Roche Diagnostics GmbH, Mannheim, Germany). Samples with a cut-off index ≥ 1.0 mIU/mL represented a reactive result. Anti-HBs detection was performed using Elecys^®^ (Roche Diagnostics GmbH, Mannheim, Germany), with a lower limit of 2 mIU/mL and an upper limit of 1000 mIU/mL. Medical notes from the Occupational Health Surveillance program were gathered along with demographic, clinical and laboratory data.

The first sample results were used to determine each participant’s long-term HBV immunity. The interval following primary vaccination was defined as the interval since date of birth (assumed to be the first vaccination day) until the date of the first sample collection. Participants were stratified into three groups based on the interval since vaccination: group 1 (16–20 years), group 2 (21–25 years) and group 3 (26–28 years).

Anti-HBs levels equal to or greater than 10 mIU/mL were considered protective, while levels that fell below 10 mIU/mL were considered insufficient for protection. A positive HBsAg test indicated hepatitis B infection [20,21,22].

Furthermore, participants with anti-HBs levels between 10 and 1000 mIU/mL at baseline and multiple follow-ups of anti-HBs levels during training or working were evaluated for the rate of decline in anti-HBs levels.

### 2.3. Statistical Analysis

Statistical analysis was performed using STATA/SE 16.1 (StataCorp LLC, College Station, TX, USA). The quantitative variables are expressed as median and interquartile range (IQR) while the qualitative variables in the data are shown as frequency and percent. The Bonferroni analysis method adjusted the alpha of 0.05 to compare the difference between groups. A mixed-model regression analysis was used to determine the declining rate of anti-HBs levels, and the Mann–Whitney U test was used to compare differences. A *p*-value < 0.05 was considered statistically significant.

## 3. Results

### 3.1. Patient Characteristics

Four hundred and seventy-eight participants met the inclusion criteria of being born after 1 January 1992, and available for anti-HBs and HBsAg testing. Fourteen participants were excluded (six participants had incomplete data, three were positive for HBsAg and five were documented as receiving a booster before anti-HBs testing). HBsAg seroprevalence was 0.6% (3/478 participants). A total of 464 participants were eligible for determination of immunogenic status. Of these, 72 (15%) were male. The median age of participants was 23 years (IQR, 22–25). Interval since first vaccination ranged from 16 to 28 years, with 16%, 69% and 15% of participants being in interval groups 1, 2 and 3, respectively. Insufficient anti-HBs levels (anti-HBs < 10 mIU/mL) were found in 51% of participants (Table 1).

### 3.2. Anti-HBs Antibody Maintenance and Duration (Years) after Primary Vaccination

The proportion of participants with anti-HBs levels < 10 mIU/mL in group 1 was 72%, significantly different from groups 2 and 3 (*p* < 0.001). However, there was no difference in proportions of patients with insufficient HBs antibodies between group 2 (48.3%) and group 3 (40%) (*p* = 0.627). The differing anti-HBs levels following primary vaccination in groups 1, 2 and 3 are presented in Table 2.

### 3.3. Changes in Anti-HBs Levels over Time

One hundred and twenty-seven participants with initial anti-HBs levels of 10–1000 mIU/mL and at least one additional follow-up result met the analysis criteria. Mixed-model regression analysis was used to determine the change in the rate of anti-HBs. Overall, anti-HBs levels decreased at a rate of −42.39 mIU/mL per year (95% CI, −54.06 to −30.73 mIU/mL; *p* < 0.001).

**Figure 1:**
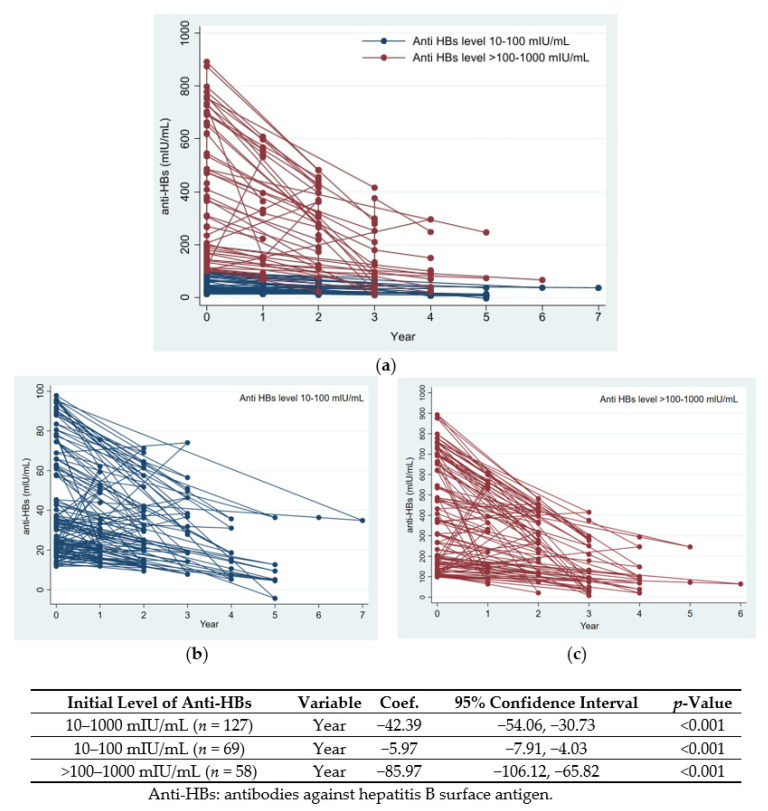
Rate of decline of anti-HBs using mixed-model regression analysis. (**a**) Anti-HBs 10–1000 mIU/mL, (**b**) anti-HBs 10–100 mIU/mL, (**c**) anti-HBs > 100–1000 mIU/mL.

Subgroup analysis revealed that the rate of decline of anti-HBs in participants with anti-HBs > 100–1000 mIU/mL was −85.97 mIU/mL per year (95% CI, −106.12 to −65.82 mIU/mL per year), whereas the rate of anti-HBs decline in those with 10–100 mIU/mL was −5.97 mIU/mL per year (95% CI, −7.91 to −4.03 mIU/mL per year) (Figure 1).

## 4. Discussion

Our retrospective study analyzed all participants born after 1 January 1992 who received an HBV vaccination as part of the EPI policy and obtained HBsAg and anti-HBs results for the first time when they began working or training in medical school. Our data showed that HBsAg seroprevalence was as low as 0.6% in vaccinated participants. These results are consistent with a previous study that reported that only 0.7% of the children born after implementation of the EPI strategy were HBsAg carriers [23].

A total of 464 participants were recruited for the study, with the majority (69%) demonstrating an interval of between 21 and 25 years from vaccination in infancy to HBV immunity testing. Sixteen and fifteen percent had time intervals of 16–20 and 26–28 years, respectively, from vaccination to HBV immunity status testing. The majority (85%) of participants were female. Regardless of the interval since vaccination, the overall seroprotection rate was 49% upon first testing. In contrast to our findings, Rao et al. and Batra et al. reported high proportions of durable HBV immunity after primary vaccination, at 89% and 70%, respectively [24,25].

Our findings are consistent with those of Posuwan et al., who discovered that more than 90% of first-year medical students (median age, 17.8 years) who received HBV vaccination according to the national protocol did not have protective levels of anti-HBs at this age [16]. Our results show that 72% (54/75) of participants in the 16–20-year interval group demonstrated anti-HBs levels of <10 mIU/mL, indicating that receiving the primary HBV vaccine series in infancy did not provide long-term immunity in all individuals.

Anti-HBs levels after vaccination declined over time. Several studies [26,27,28] found a negative correlation between anti-HBs titers and time. However, our findings revealed that the highest rate of antibody loss was 72% in individuals with a shorter duration since vaccination (group 1, 16–20-year interval), compared with that in individuals with a longer duration since vaccination (48.3% in group 2 and 40% in group 3). Previous studies have revealed that the durability of the anti-HBs response was also related to age at vaccination and peak antibody response achieved. By 18 years post-vaccination, 16% of persons vaccinated at age < 1 year have detectable antibodies, compared with 74% of those vaccinated at age > 1 year [22]. Our results may be explained by the lag time in the distribution of HBV vaccine during the early phase of the EPI rollout in Thailand; thus, participants in groups 2 or 3 may have received the complete vaccination course after the infancy period or in childhood. Additionally, natural boosters from occupational risk, subclinical breakthrough HBV infection and unreported boosters in childhood are all factors that can contribute to the establishment of hepatitis B immunity later in life. Due to their older age at the time of the initial anti-HBs determination, the age groups 21–25 and 26–28 years post-primary immunization have a higher risk of occupational exposure. Anti-HBc testing to determine breakthrough infection was not performed on a regular basis at our institution. However, research by Yong et al. found that out of 109 newborns who received primary immunization, 24 (22%) showed strong serological evidence of natural HBV exposure over a 20-year follow-up period. In 10% of participants in the first decade and 10.7% of subjects in the second decade, there was an increase in anti-HBs concentration that was unrelated to extra HBV vaccination. These findings are most likely the result of natural boosting. The majority of these findings were found in subjects who were born to mothers who were HBsAg-positive [29], which along with the eras of 21–25 and 26–28 years post-primary immunization had a higher incidence of HBsAg-positive in mothers than the 16–20-year post-primary immunization age group.

Additionally, a recombinant DNA vaccine (Engerix-B) comprising HBV genotype A2 and HBsAg subtype adw2 was one of the frequently used vaccines in Thailand. According to Stramer et al., this form of the vaccine may provide less protection against HBV nonA2 genotype infection [30].

Despite the loss of anti-HBs antibodies or a decline in anti-HBs levels below 10 mIU/mL, there is evidence of long-lasting cellular immunity, implying that the protection provided by primary immunization throughout childhood and adulthood lasts for 32 years [31]. Additionally, 88% of individuals who had been post-primary vaccinated for 30 years and had an anti-HBs level of <10 mIU/mL responded to a booster dose with an anti-HBs level of ≥10 mIU/mL at 30 days, demonstrating durable immunity to HBV infection [32].

However, Doi et al. [17] published an article describing the factors that influence the persistence of hepatitis B vaccine responses. Regarding anti-HBs antibody maintenance and booster vaccination, they defined that when anti-HBs antibody titers decline, a risk of HBV infection arises because decreased neutralizing antibody titers may be insufficient to completely prevent HBV infection [30]. The hepatitis B vaccine strategy effectively prevents infection with HBV and its complications, thus maintaining an appropriate level of anti-HBs may be beneficial for ensuring the safety of healthcare providers. An HBV vaccine schedule should be offered to HCWs and anti-HBs levels should be monitored annually or periodically, with booster vaccines administered according to anti-HBs levels.

As a result, the most appropriate approach for management in HCWs remains in question. There is an argument for booster HBV vaccination for individuals who have completed the three-dose series of HBV vaccine and subsequently been documented with anti-HBs < 10 mIU/mL. According to the Centers for Disease Control and prevention, such responders do not require a booster, despite the risk of accidental exposure or the requirement for annual anti-HBs testing, except for hemodialysis patients [22]. Global hepatitis B incidence is wide ranging, with the Sub-Saharan African and Western Pacific regions classified as having a high-intermediate–high endemicity of hepatitis B (at least 5%–≥8% prevalence, perhaps higher than 15%). The European and Eastern Mediterranean regions had low–intermediate HBV infection rates (2–4.99%), while the North Americas and Western Europe were low-endemic regions, with an HBsAg prevalence of less than 2% [33,34,35]. It is questionable whether a similar approach is reasonable in areas of intermediate–high prevalence of HBV. Breakthrough infection among vaccinated persons with undetectable anti-HBs was also reported [36]. The presence of occult HBV infection may be associated with an increased risk of HCC or chronic liver disease. Therefore, there is a view that if anti-HBs levels fall below 10 mIU/mL, annual anti-HBs testing and booster doses should be considered for those with an ongoing risk of exposure [37].

For this approach to be the most cost-effective, we investigated the rate of decline of HBV immunity to predict the duration of vaccine-induced seroprotection and optimal timing for anti-HBs testing. Our study demonstrated that the overall rate of decline in anti-HBs was −42.39 mIU/mL per year. The rate of anti-HBs decline varied by initial anti-HBs level. Participants with protective immunity of anti-HBs > 100–1000 mIU/mL had a rapid decline in anti-HBs levels with a rate of −85.97 mIU/mL per year, whereas those with anti-HBs of 10–100 mIU/mL had a rate of decline of −5.97 mIU/mL. The results of our study provide guidance for determining the optimal period for rechecking the HBV immune status of HCWs.

The kinetics of anti-HBs in the group of anti-HBs > 100–1000 mIU/mL was rapid decay compared with the group of anti-HBs 10–100 mIU/mL. Although all participants in our study were not documented for booster vaccination before or during anti-HBs level monitoring, this pattern of antibody decline was concordant with Verso et al.’s study [38]. They reported that nurse students who had received a booster vaccination showed an exponential reduction in anti-HBs titers, whereas those who did not receive the booster showed a linear reduction. The natural booster from exposure to HBV, such as occupational exposure and sexual contact, could be considered; however, we did not have any data for those events.

Another study from Japan [39] also demonstrated the acquisition rate of antibody decline among 832 medical and dental students. The relationship between antibody titer at one month (x) and five months (y) was estimated by log_10_y = log_10_x − 0.134, indicating that the anti-HBs titer decreased by an average of 20% within four months.

Our study had some limitations. Firstly, because of the retrospective nature of this study, medical records may not contain all of the necessary information, such as the backgrounds of HCWs. Secondly, serial testing for antibody levels was not performed in all participants. Lastly, the small sample size enrolled in this study may also affect the results. A large-sample prospective study should be conducted to provide additional information.

## 5. Conclusions

Our study highlighted participants’ long-term immunity following primary hepatitis B vaccination in a high-prevalence area. Additionally, we determined the rate of decline of HBV immunity in subgroups with higher and lower anti-HBs levels. Immunity to HBV deteriorates over time. Furthermore, the rate of HBV decline is affected by the initial anti-HBs level achieved. As a national policy, the infant hepatitis B immunization program may not provide lifelong immunity to HBV and therefore HCWs may benefit from regular monitoring of immune status.

## Figures and Tables

**Table 1:** Clinical characteristics of participants (*n* = 464).

Characteristics	*n* (%)
Median age, years (IQR 25th–75th percentile)	23 (22–25)
Male	72 (15)
Time since primary vaccination (years)	
16–20	75 (16)
21–25	319 (69)
26–28	70 (15)
Initial level of anti-HBs (mIU/mL)	
<2	167 (36)
2–<10	69 (15)
10–1000	178 (38)
>1000	50 (11)

Anti-HBs: antibodies against hepatitis B surface antigen, IQR: interquartile range.

**Table 2:** Antibodies against hepatitis B surface antigen status after primary vaccination (years).

Duration after Primary Vaccination (Years)	*n*	Number of Participants with Initial Anti-HBs Level, *n* (%)			
		<10 (mIU/mL)		10–1000 (mIU/mL)	>1000 (mIU/mL)
		<2 (mIU/mL)	2–<10 (mIU/mL)		
Group 1: 16–20	75	32 (42.7)	22 (29.3)	19 (25.3)	2 (2.7)
Group 2: 21–25	319	112 (35.1)	42 (13.2)	124 (38.9)	41 (12.8)
Group 3: 26–28	70	23 (32.9)	5 (7.1)	35 (50)	7 (10)

Anti-HBs: antibodies against hepatitis B surface antigen.

## Data Availability

The datasets generated and analyzed during the current study are available from the corresponding author upon reasonable request.
